# In vivo assessment of mitral valve leaflet remodelling following myocardial infarction

**DOI:** 10.1038/s41598-022-22790-0

**Published:** 2022-10-26

**Authors:** Bruno V. Rego, Amir H. Khalighi, Eric K. Lai, Robert C. Gorman, Joseph H. Gorman, Michael S. Sacks

**Affiliations:** 1 James T. Willerson Center for Cardiovascular Modeling and Simulation, Oden Institute for Computational Engineering and Sciences, and the Department of Biomedical Engineering, The University of Texas at Austin, Austin, TX USA; 2 Gorman Cardiovascular Research Group, Department of Surgery, Perelman School of Medicine, University of Pennsylvania, Philadelphia, PA USA

**Keywords:** Interventional cardiology, Experimental models of disease

## Abstract

Each year, more than 40,000 people undergo mitral valve (MV) repair surgery domestically to treat regurgitation caused by myocardial infarction (MI). Although continual MV tissue remodelling following repair is believed to be a major contributor to regurgitation recurrence, the effects of the post-MI state on MV remodelling remain poorly understood. This lack of understanding limits our ability to predict the remodelling of the MV both post-MI and post-surgery to facilitate surgical planning. As a necessary first step, the present study was undertaken to noninvasively quantify the effects of MI on MV remodelling in terms of leaflet geometry and deformation. MI was induced in eight adult Dorset sheep, and real-time three-dimensional echocardiographic (rt-3DE) scans were collected pre-MI as well as at 0, 4, and 8 weeks post-MI. A previously validated image-based morphing pipeline was used to register corresponding open- and closed-state scans and extract local in-plane strains throughout the leaflet surface at systole. We determined that MI induced *permanent* changes in leaflet dimensions in the diastolic configuration, which increased with time to 4 weeks, then stabilised. MI substantially affected the systolic *shape* of the MV, and the *range of stretch* experienced by the MV leaflet at peak systole was substantially reduced when referred to the current time-point. Interestingly, when we referred the leaflet strains to the pre-MI configuration, the systolic strains remained very similar throughout the post-MI period. Overall, we observed that post-MI ventricular remodeling induced permanent changes in the MV leaflet shape. This predominantly affected the MV’s diastolic configuration, leading in turn to a significant decrease in the range of stretch experienced by the leaflet when referenced to the current diastolic configuration. These findings are consistent with our previous work that demonstrated increased plastic (i.e. non-recoverable) leaflet deformations post-MI, that was completely accounted for by the associated changes in collagen fiber structure. Moreover, we demonstrated through noninvasive methods that the state of the MV leaflet can elucidate the progression and extent of MV adaptation following MI and is thus highly relevant to the design of current and novel patient specific minimally invasive surgical repair strategies.

## Introduction

The mitral heart valve (MV) valve regulates blood flow between the left atrium and left ventricle (LV). Due to its intimate anatomical integration with the LV via the annulus, chordae tendineae (MVCT), and papillary muscles (PMs), the MV is considered part of the LV functional unit. Ischaemic MV regurgitation (IMR) often follows a myocardial infarction (MI), whose aftereffects distort the MV geometry through annular dilation and MVCT tethering, and the severity of IMR coincident with LV dysfunction has been directly associated with premature death^1,2^. More broadly, the societal burden of MI-induced IMR is expected to continue to increase, given existing trends in the growth of the elderly population^2^.

While IMR has most successfully been addressed through a surgical MV repair procedure known as undersized ring annuloplasty (URA)^3^, in which the annular orifice area is forcibly decreased, this approach remains problematic, with about a third of repairs failing over the longer term (i.e. leading to a recurrence of IMR)^4^. It has been hypothesised that the failure rate of URA-based repairs is at least partially attributable to the substantial changes in MV geometry and closing behaviour that this procedure induces, including constriction and flattening of the annulus^5,6^. Moreover, the repair does not halt continued LV remodelling post-MI, which over time alters the MV’s “boundary conditions” sufficiently to cause recurrent IMR^7–9^. The mechanisms of MV repair failure have been partly elucidated through analysis of real-time three-dimensional echocardiography (rt-3DE) images collected pre- and post-surgery. Recently, Bouma et al. showed that the pre-surgical tethering angle of the posterior leaflet could be used as a predictor of recurrent IMR 6 months after URA^10^. Our current knowledge of MV function and IMR-induced remodelling thus suggests that despite high rates of short-term repair success, in some cases URA may actually exacerbate posterior leaflet tethering by displacing the posterior annulus anteriorly, which increases the probability of recurrent IMR over the long term^10,11^.

Accurate prediction of MV repair success or failure, for the purposes of patient stratification and/or personalised treatment, will depend on developing a fuller understanding of the interrelationships between the MV’s geometry, mechanics, and capacity for biologically mediated remodelling. The accumulated body of previous studies relating particular preoperative MV characteristics (e.g. annular shape, tethering) to the success or failure of URA underscores the possibility and an urgent clinical need for noninvasive, patient-specific methods to optimise MV repair procedures based on pre-surgical information alone^10–14^. In addition, post-MI and post-URA MV growth and remodelling undoubtedly play a critical role in determining repair outcomes^15^. In addition to changes in tissue-level properties, there is evidence of MV interstitial cell activation and matrix turnover in the MV leaflets following MI^16,17^. Due to the established causal link between tissue stretch and cell-driven remodelling mechanisms, a detailed account of tissue-level deformation patterns in the post-MI MV can provide substantial insight into the current biosynthetic state of the MV^18,19^. Consequently, it is likely that quantitative estimates of MV leaflet tissue deformation, and changes therein following MI, can serve as valuable independent predictors of long-term remodelling phenomena that affect MV function and thus repair outcomes. Yet, our understanding of MV tissue remodelling remains profoundly limited in both the unmodified pathological and post-surgical scenarios.

The objective of the present investigation was thus to gain insight into the post-MI MV remodelling behaviour by directly quantifying the effects of MI on MV in vivo leaflet tissue deformation. To this end, we utilised a recently developed image-based computational modelling pipeline^20^ to noninvasively quantify how the in vivo diastolic and systolic MV deformation patterns change after MI, in an effort to more fully understand how and to what extent the MV remodels post-MI. Such an understanding is critical and must be established prior to any endeavour to optimise MV repair approaches on a patient-specific basis.

## Results

**Figure 1:**
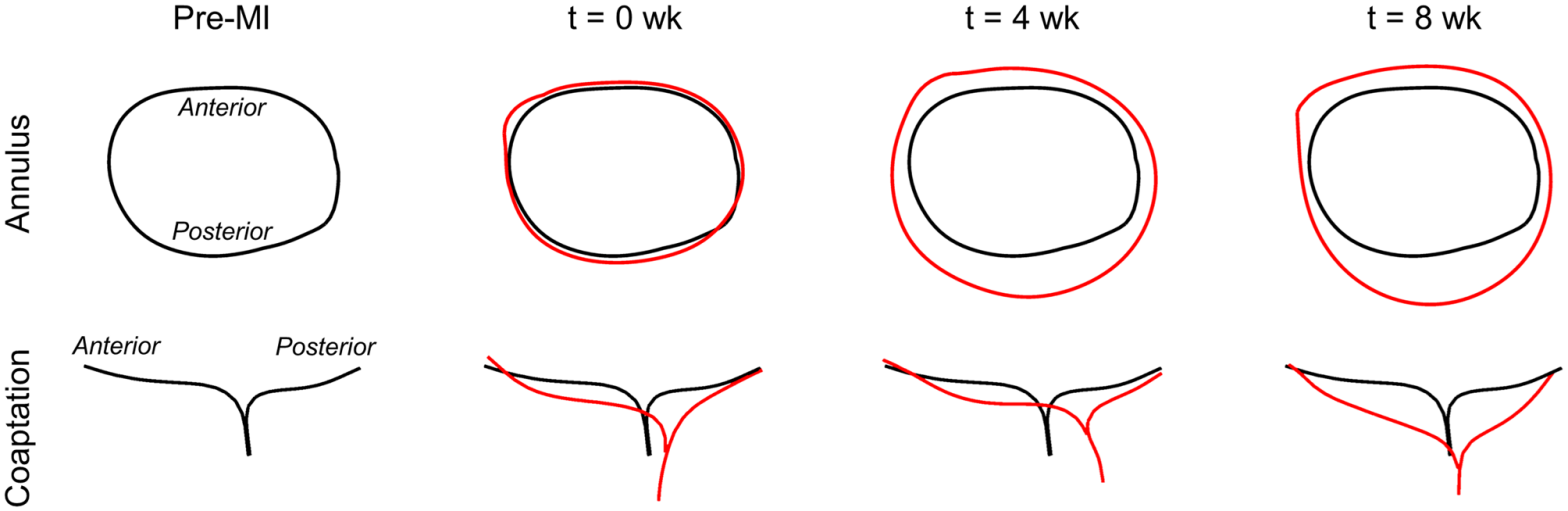
Post-MI changes in annulus shape and leaflet coaptation behaviour over time (pre-MI shown in black, post-MI shown in red). At $t = 4$ week and $t = 8$ week, both annular dilation and leaflet tethering are substantial but appear to have begun to stabilise.

**Figure 2:**
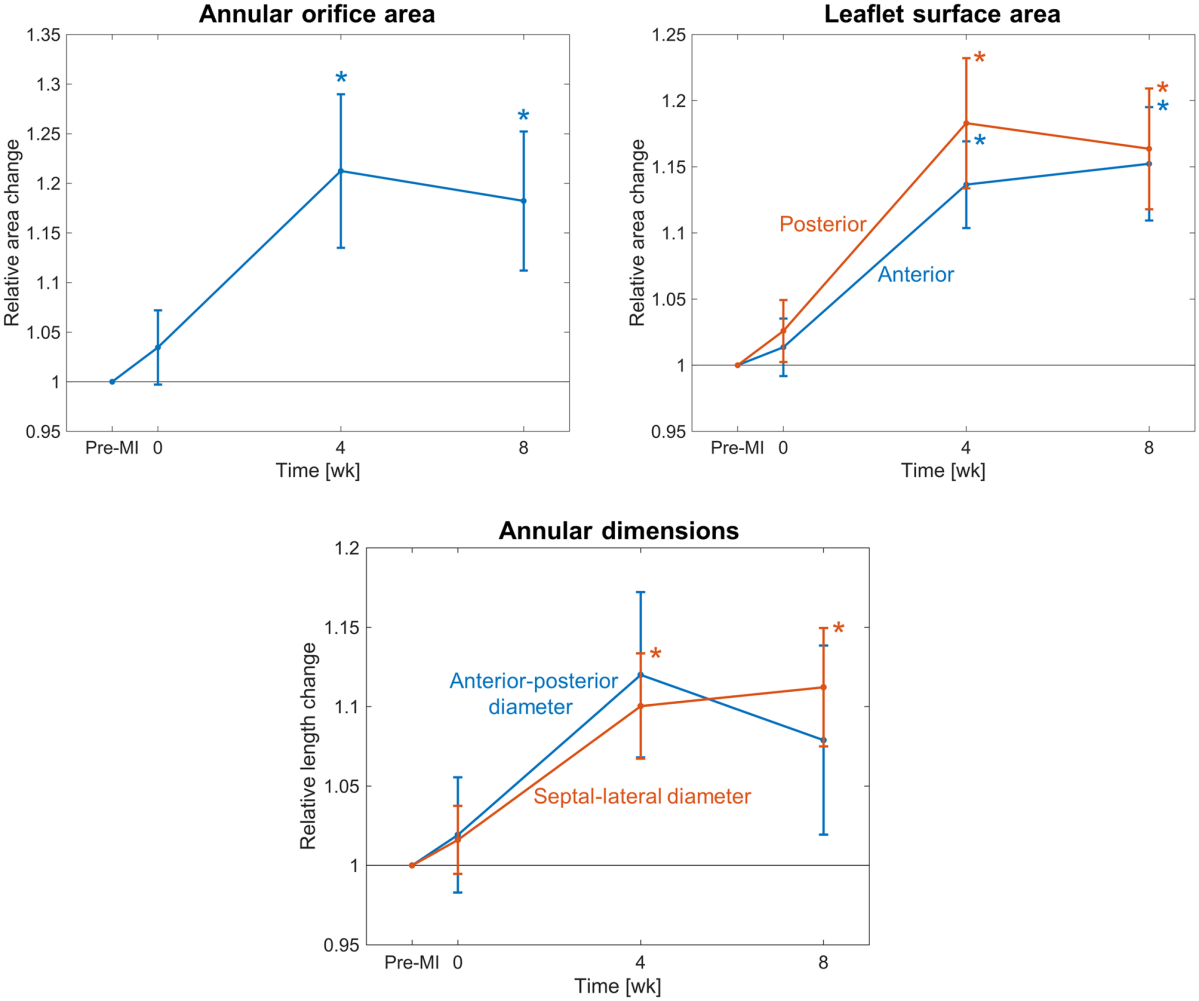
Changes in annular orifice area, the surface area of each leaflet, and annular dimensions over time, relative to their respective pre-MI values. Qualitatively, annular dilation and enlargement of both leaflets move in tandem, all increasing substantially within 4 weeks post-MI and apparently stabilising by 8 weeks post-MI.

Initial analysis of rt-3DE images from eight ovine subjects collected pre-MI as well as 0, 4, and 8 weeks post-MI indicated that the MV annulus became substantially dilated post-MI, consistent with previous findings (Fig. 1)^21^. Moreover, both leaflets were severely tethered post-MI. Paired $t$-tests revealed that at $t = 4$ weeks and $t = 8$ weeks, relative changes in both annular orifice and leaflet surface areas were significantly greater than their pre-MI values, with average increases of 20% and 16% respectively (Fig. 2). The increase in annular orifice area was associated with dilation along both the anterior-posterior and septal-lateral directions, in roughly equal proportion. Interestingly, no significant differences were found between 4- and 8-week values of any dimensional or areal metric for the annulus or leaflets. In addition, the relative increase in anterior and posterior leaflet surface areas were not found to be different at any time point, suggesting that the two leaflets remodel at about the same rate (Fig. 2).

**Figure 3:**
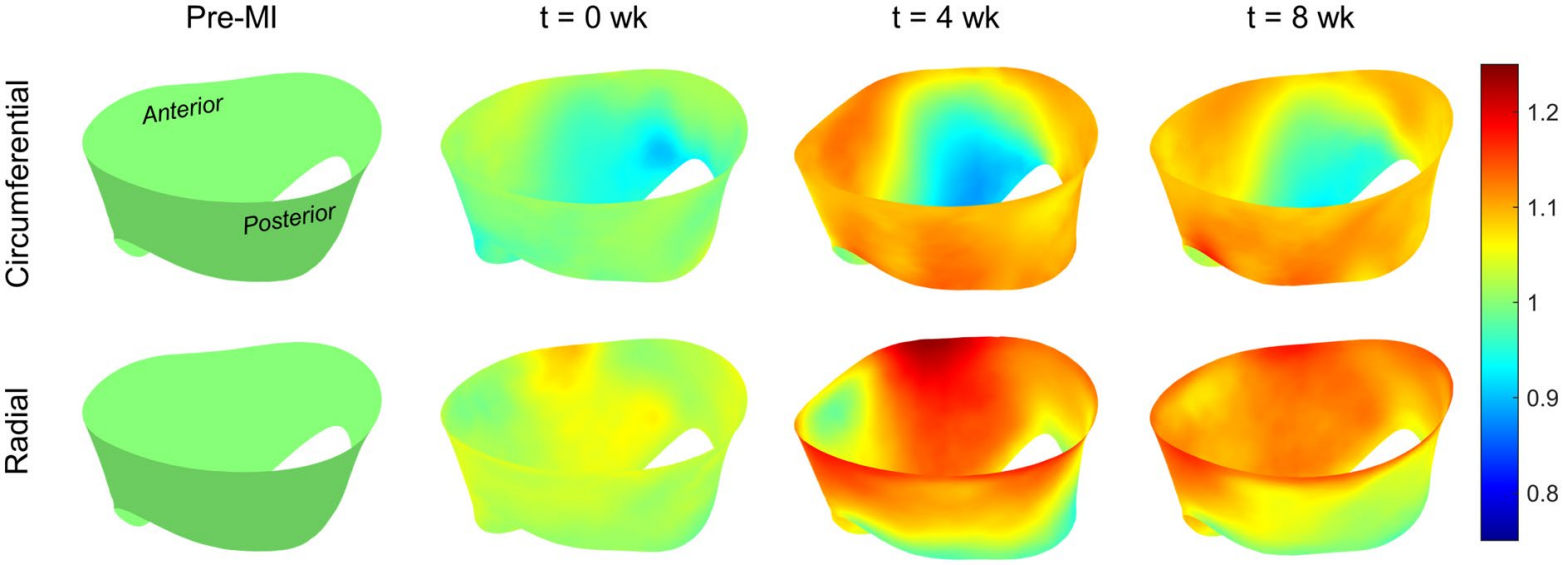
3D MV geometry showing the average diastolic permanent directional stretch fields at each post-MI time point, referenced to the pre-MI diastolic configuration. As the post-MI remodelling process continued, the MV underwent increasing plastic deformation, with further increases ceasing after 4 weeks. These “deformation” patterns are a result of permanent changes in the opened shape of the MV, and should be distinguished from systolic strains that are driven by systolic pressure when the MV is closed.

These MI-induced changes in MV geometry propagated throughout both leaflets over time, yielding substantial changes in their in vivo leaflet diastolic shape and systolic stretch patterns. First, we observed the presence of substantial *permanent leaflet deformations* in the diastolic (fully opened) state. These deformations were permanent in the sense that they appeared in the diastolic (opened) configuration, which is under minimal loading. Moreover, such permanent changes in the MV leaflet have been observed in previous studies on post-MI excised leaflets using the same animal model^18,22^. As such, they are not associated with other mechanisms and represent intrinsic changes to the MV leaflet dimensions. We thus quantified these permanent changes as a form of plastic deformation, represented as stretches in the circumferential and radial directions referenced to the initial pre-MI configuration (see the pre-MI state in Fig. 1). Results of this analysis indicated substantial plastic stretches in both the circumferential and radial directions, with significant regional heterogeneity (Fig. 3). Moreover, these changes appeared to stabilise after 4 weeks, as evidenced by the similarities between the 4 and 8 week data (Fig. 3).

**Figure 4:**
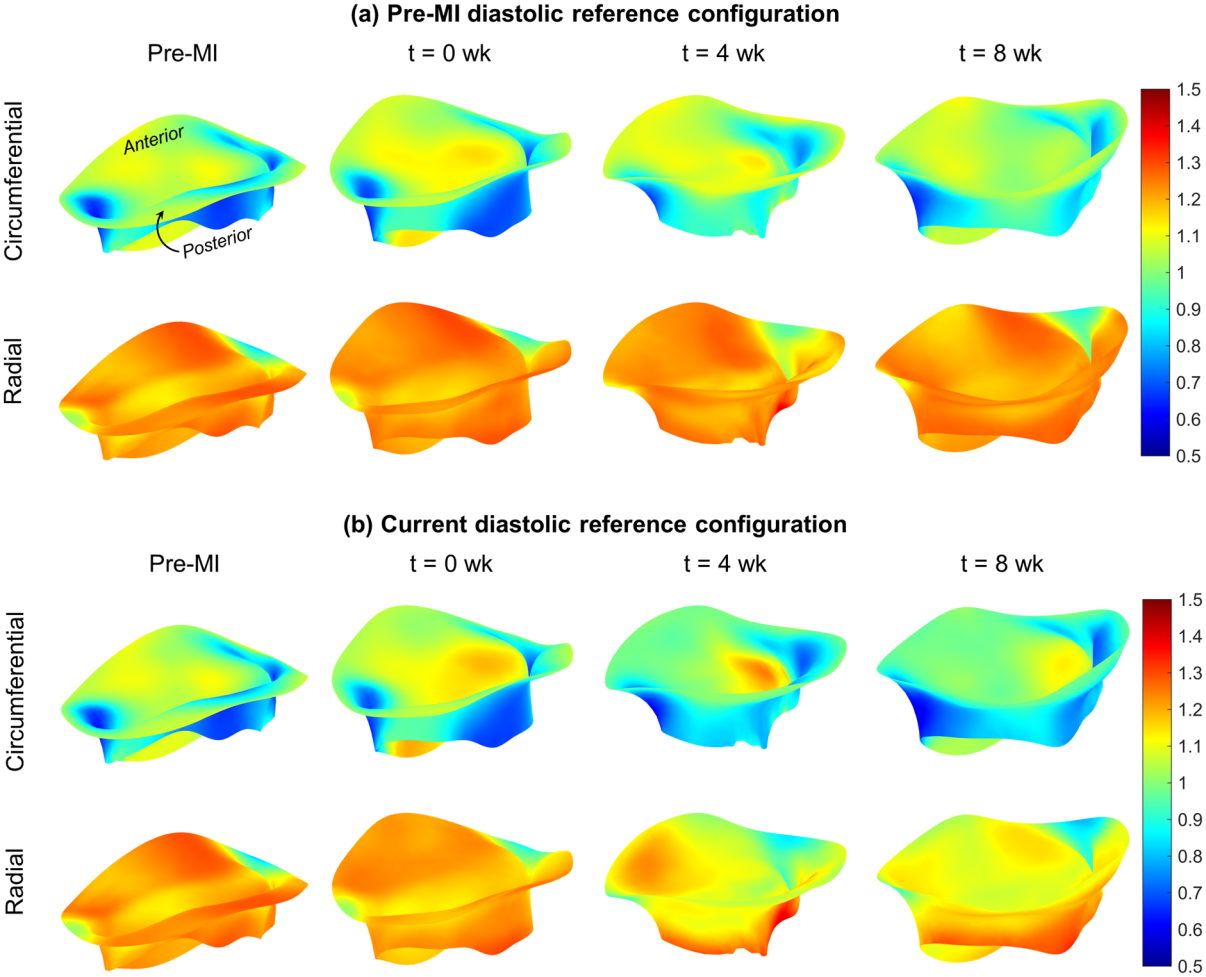
Average systolic directional stretch fields over time, referenced to (**a**) the pre-MI diastolic configuration and (**b**) the current time point’s diastolic configuration. The results in (**a**) show negligible changes in both directions over time, suggesting that the fibre recruitment behaviour of the leaflet tissue remains largely the same post-MI in an absolute sense. The results in (**b**) show that the range of stretch experienced by the leaflets during the cardiac cycle is substantially decreased over time, specifically in the radial direction. This decrease can be attributed to the pre-stretch already experienced by the leaflets in diastole, which consumes much of the tissue’s overall extensibility. See Discussion for further interpretation.

**Figure 5:**
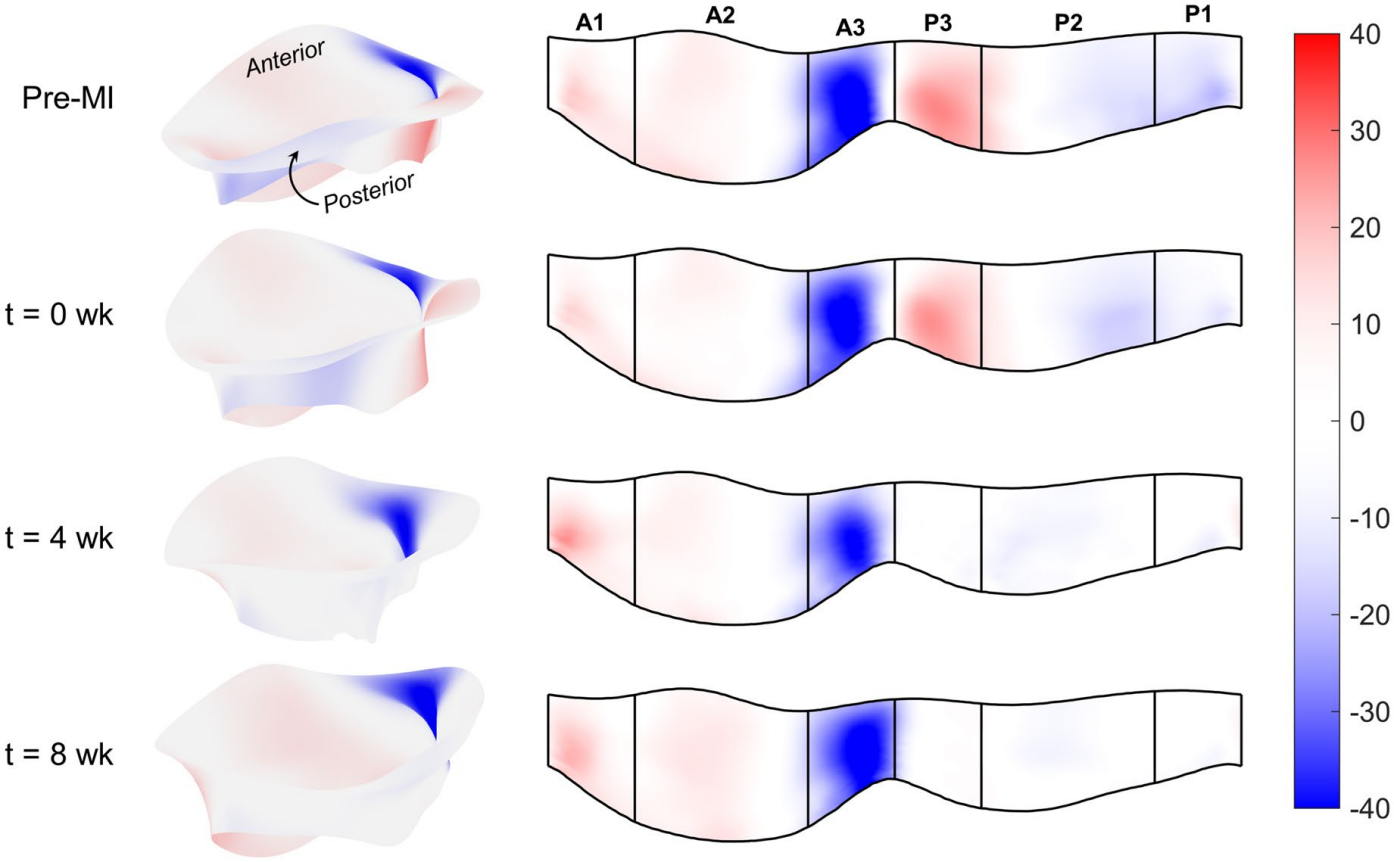
Average systolic shear angle fields over time (in degrees), shown on both the 3D and 2D parametric representations of the leaflet surface. 3D surfaces are shaded for visualisation. Note that by 4 weeks post-MI, the magnitude of shearing in the posterior leaflet becomes negligible. Diastolic shear angle magnitudes were not substantial for any time point (Figure S4), and are thus not shown here (see Supplementary Information for diastolic and systolic shear angle maps in 2D).

We next examined the systolic stretch behaviours post-MI, which result exclusively from systolic loading. Based on the observations of time-evolving diastolic reference configurations, we utilized both the pre-MI and current diastolic reference configurations in our computations. When referenced to the *pre-MI diastolic reference configuration*, the directional stretches remained largely unchanged over much of the leaflet (Fig. 4a). Interestingly, when referenced to the *current diastolic state*, the magnitudes of the systolic stretches indicated a gradual diminution in magnitude over the 8 weeks time period (Fig. 4b). Post-MI changes in deformation also altered shear patterns in certain regions of the leaflet, significantly decreasing the magnitude of the shear angle experienced by the posterior leaflet commissural segments (P1, P3) in systole (Fig. 5).

**Figure 6:**
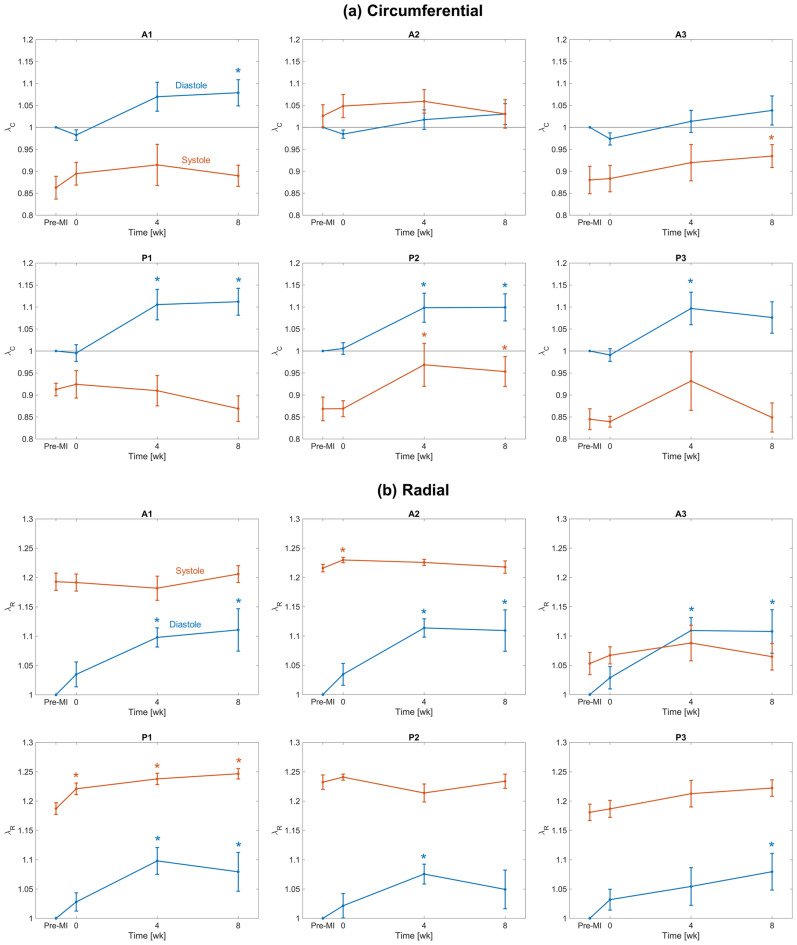
(**a**) Circumferential and (**b**) radial stretches in both diastole and systole for each Carpentier leaflet segment, referenced to the pre-MI diastolic configuration. Lines and error bars at each time point denote the mean and standard error across $n = 8$ specimens. Asterisk symbols (*) denote statistically significant differences from the corresponding pre-MI value with 95% confidence ($p < 0.05$ yielded by a paired $t$-test).

**Figure 7:**
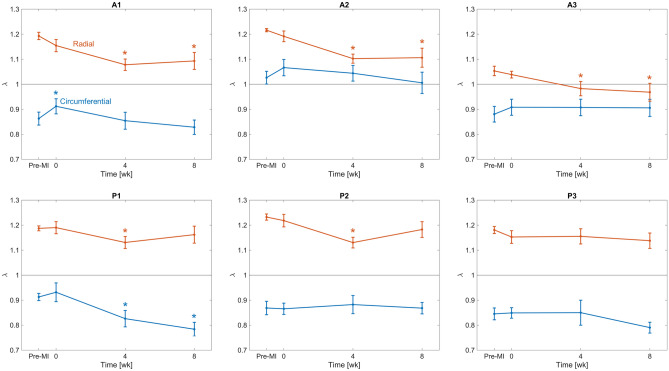
Systolic stretches in both the circumferential and radial directions, referenced to the current time point’s diastolic configuration. Lines and error bars at each time point denote the mean and standard error across $n = 8$ specimens. Asterisk symbols (*) denote statistically significant differences from the corresponding pre-MI value with 95% confidence ($p < 0.05$ yielded by a paired $t$-test). While results in Figs. 4a and 6 demonstrate that the systolic configuration of the MV actually remains largely unchanged post-MI, the results above highlight that the radial deformation experienced by the leaflets over the current cardiac cycle is significantly decreased throughout several regions of the leaflet, due to the effects of tethering that stretch out the leaflets in diastole over time.

**Figure 8:**
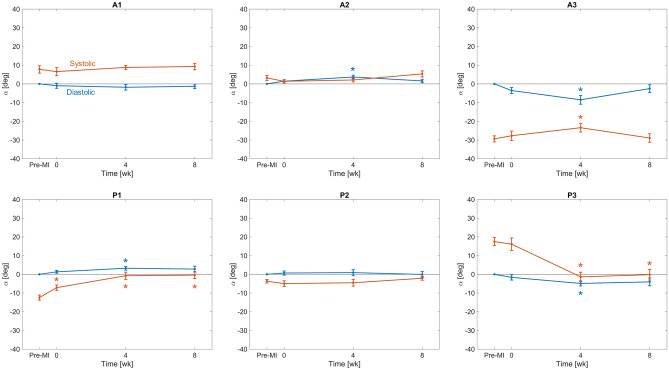
Shear angle in both diastole and systole for each Carpentier leaflet segment, referenced to the pre-MI diastolic configuration. Lines and error bars at each time point denote the mean and standard error across $n = 8$ specimens. Asterisk symbols (*) denote statistically significant differences from the corresponding pre-MI value with 95% confidence ($p < 0.05$ yielded by a paired $t$-test).

Next, to clarify these findings as regional trends, we computed the mean stretch values for each Carpentier leaflet segment (see "Methods" for details). When referenced to the pre-MI diastolic state, continued increases in diastolic circumferential stretches were observed in all posterior segments at 4 weeks, which generally stabilised thereafter (Fig. 6a). In contrast, the systolic stretches were compartively stable, with statistically significant but small in magnitude changes which again stabilised after 4 weeks (Fig. 6a). Similar trends were observed in the radial direction, with generally more pronounced changes in the diastolic configuration (Fig. 6b). In contrast, when the current diastolic referential configuration was utilized, the resulting systolic stretches indicated marked reductions in both the circumferential and radial directions, especially in the anterior leaflet (Fig. 7). Statistical analyses supported these findings (Table 1). Shear strain analyses generally revealed negligible changes in the A1, A2, and P2 segments (Fig. 8). In contrast, the P1 and P3 segments demonstrated gradual reductions in systolic shear strains, which essentially vanished by 4 weeks. Collectively, these important results suggest that the MV’s post-MI remodelling is driven mostly by alterations in its diastolic configuration.

**Table 1:** Qualitative changes in directional stretches within the central regions of the anterior and posterior leaflet (segments A2 and P2). Arrows indicate whether the corresponding stretch is increased or decreased relative to its pre-MI value. Statistical significance was determined using a paired $t$-test for each segment, with a 95% confidence level.

Direction	Leaflet	Cardiac cycle phase	Time post-MI [weeks]		
			0	4	8
Circumferential	Anterior	Diastolic	–	–	–
		Systolic (w.r.t. pre-MI diastolic)	–	–	–
		Systolic (w.r.t. current diastolic)	–	–	–
	Posterior	Diastolic	–	$\uparrow$	$\uparrow$
		Systolic (w.r.t. pre-MI diastolic)	–	$\uparrow$	$\uparrow$
		Systolic (w.r.t. current diastolic)	–	–	–
Radial	Anterior	Diastolic	–	$\uparrow$	$\uparrow$
		Systolic (w.r.t. pre-MI diastolic)	$\uparrow$	–	–
		Systolic (w.r.t. current diastolic)	–	$\downarrow$	$\downarrow$
	Posterior	Diastolic	–	$\uparrow$	–
		Systolic (w.r.t. pre-MI diastolic)	–	–	–
		Systolic (w.r.t. current diastolic)	–	$\downarrow$	–

## Discussion

Computational models of the MV have greatly improved our understanding of MV biomechanics and mechanobiology^23–26^. Moreover, through the combination of experimental and computational approaches, we and others have made substantial strides in elucidating how the MV tissue microarchitecture governs its organ-level behaviour, and how these might be comprehensively investigated in pathological contexts, such as IMR^15,20,25,27–35^. Such advances in computational modelling allow quantitative assessment of the progression of MI-induced remodelling and disease^36^. Ultimately, these approaches will enable the identification of optimal therapies for IMR on a patient-specific basis, thus paving the way toward personalised MV repair and stratification of patients into MV repair and replacement groups.

However, enabling such models requires a detailed understanding of MV remodelling, of which there remains a paucity of information. In the present study, we have taken a step towards addressing this issue utilising our noninvasive image-based computational method to quantify MV leaflet deformations. We divided our analysis into two functional parts: (1) Changes in the open (diastolic) configuration, which are driven by plastic (i.e. non-recoverable) deformations induced by changes in the post-MI LV, such as annular dilation and leaflet tethering, and (2) changes in the closed (systolic) deformations which are induced by LV pressure. We examined how these quantities evolved during an 8-week post-MI period in the absence of treatment. As such, this study represents an essential “baseline” investigation of MV remodelling post-MI. It offers a detailed picture of the extent to which the MV is *capable* of remodelling in response to MI, even over a relatively short time. Our results bring to light several key observations with regard to post-MI MV remodelling, which include the following:

1. The LV in the post-MI state induced *permanent* increases in MV leaflet size as evidenced by the increased circumferential and radial diastolic stretches (Figs. 3, 6). As stated earlier, while we refer to these quantities as “plastic stretches” since they actually represent changes in leaflet shape not associated with any external loading. These observations are supported by our extensive publications on the remodelling events that follow IMR^17,22,37,38^. Specifically, we demonstrated that the MV leaflet stiffness is substantially higher by 4 weeks post-MI as a result of altered MV dimensions. Gross geometric and mechanical changes were accompanied by alterations in the MV collagen fibre structure but not collagen fibre elastic modulus, which maintained a value of $\sim$200 MPa. This latter finding indicated no apparent damage to the collagen fibres themselves, and that all changes were completely explained by plastic changes to the MV unloaded configuration. As these measurements were performed on excised MV leaflet tissues, they can only be due to actual intrinsic changes in the leaflet and not alterations in diastolic state boundary conditions.
2. In contrast to the diastolic state, in the post-MI systolic state the stretch experienced by the MV leaflet was substantially reduced (Figs. 4b, 7) when the stretch was referred to the *current diastolic state*.
3. When referenced to the *pre-MI diastolic state*, the post-MI systolic stretches experienced remained relatively constant (Figs. 4a, 6).
4. All changes in plastic deformation appeared to have ceased by 4 weeks.

Collectively, these results indicate that MV leaflet tissues have a significant but limited ability to undergo plastic-like deformations post-MI, which is essentially exhausted by 4 weeks. These changes are driven by the altered boundary loading (annular dilation and MVCT tethering), and do not appear to be due to any other pathophysiological processes.

In regard to related studies, it is possible to compare our findings to those of Rausch et al.^39^, who previously performed a detailed fiducial marker-based analysis of acute and chronic MV leaflet enlargement using the same ovine model of posterior MI. Although the two studies partitioned the leaflet differently for regional analysis, the results are generally in very close agreement. Specifically, both studies found that (1) immediately post-MI, diastolic stretches were not significant in either the circumferential or radial directions; (2) chronically, circumferential stretches were close to 5% on average, with significant increases detected near the anterolateral commissure (segment A1) but no differences detected near the posteromedial commissure (segment A3); (3) chronically, circumferential stretches near the free edge tended to be smaller than in the central belly, while those near the annulus tended to be larger than in the central belly; and (4) chronically, radial stretches were significant and close to 10% in the central belly region (segment A2). The agreement of these key findings serves as a notable additional validation of our novel noninvasive method for image-based stretch estimation.

**Figure 9:**
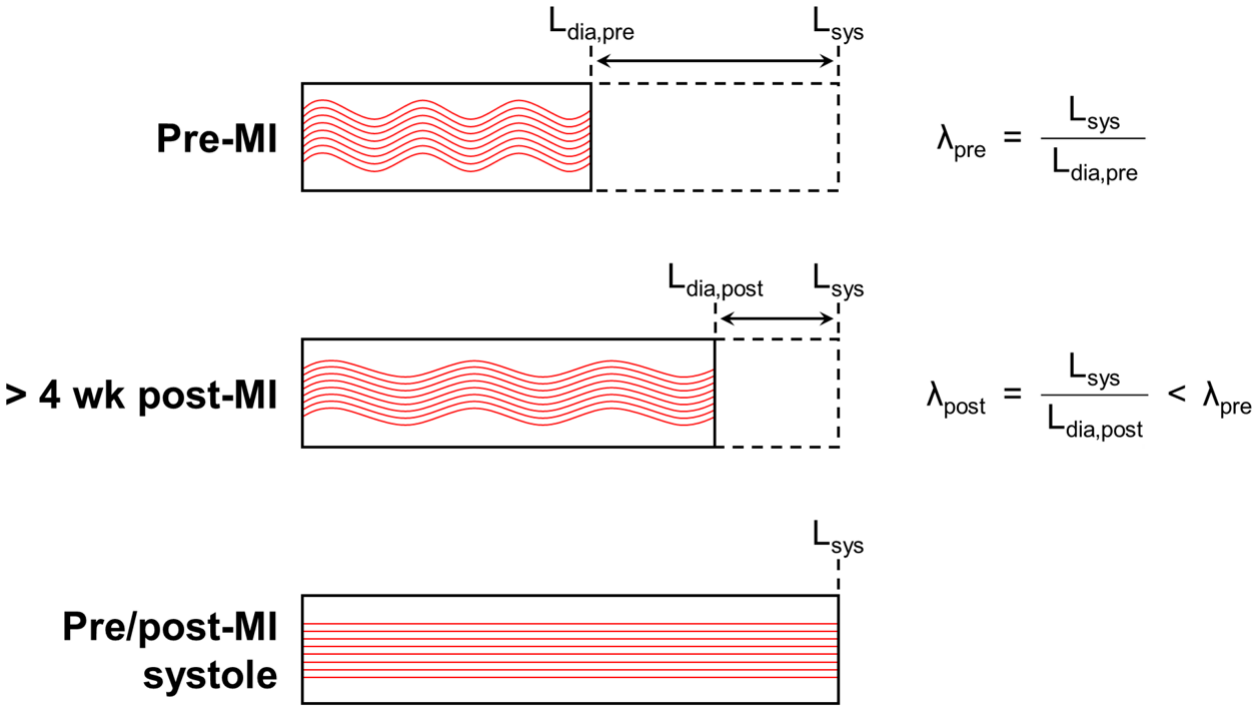
Uniaxial schematic of MV leaflet configurations that were considered in the present study, with red curves depicting how the collagen fibre geometry within the tissue would evolve with stretch (not to scale). Reference lengths for pre-MI diastole ($L_\text {dia,pre}$), post-MI diastole ($L_\text {dia,post}$), and systole ($L_\text {sys}$) are labelled, as well as systolic stretches with respect to pre-MI and post-MI diastolic configurations ($\lambda _\text {pre}$ and $\lambda _\text {post}$, respectively). Note that $L_\text {dia,post} > L_\text {dia,pre}$ and a constant $L_\text {sys}$ imply that $\lambda _\text {post} < \lambda _\text {pre}$, even in the absence of active remodelling in the collagen fibre network. Our results (especially Fig. 4a) thus suggest that active remodelling over the 8-week study period was not sufficient to effect substantial changes in the mechanical properties of the tissue. Equivalently, the *apparent* tissue stiffening observed over most of the leaflet (Figs. 4b, 10b) can be explained as a passive consequence of the MI-induced diastolic deformations (Fig. 3).

**Figure 10:**
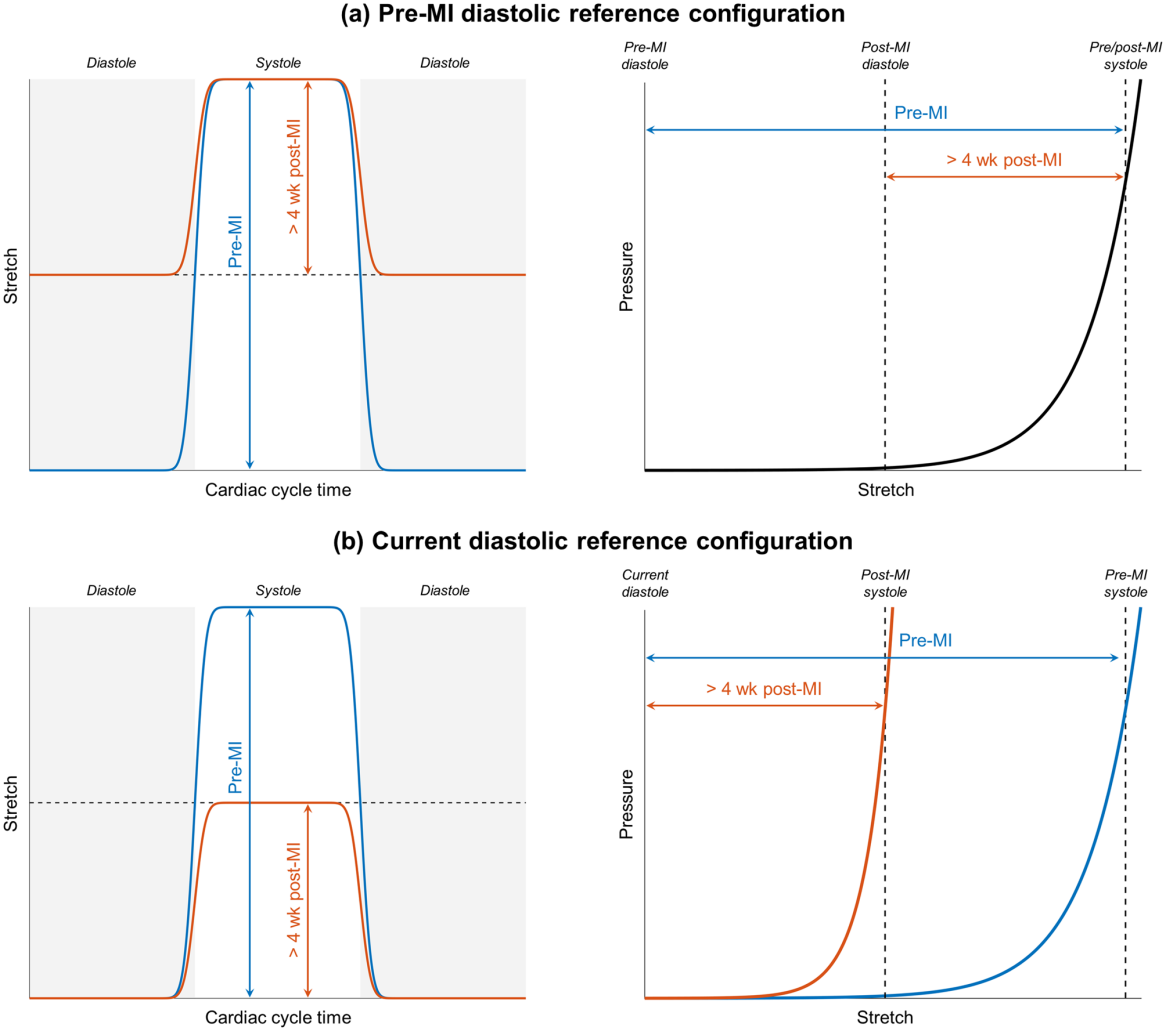
Schematic pressure waveforms and mechanical responses of the MV, referenced to (**a**) the pre-MI diastolic configuration and (**b**) the current time point’s diastolic configuration. While the leaflet’s total stretch in systole remains essentially constant (**a**), the range of stretch experienced over the cardiac cycle is substantially narrowed due to permanent diastolic distention, especially in the radial direction (Figs. 3, 6). This causes the leaflet tissue to behave more stiffly with respect to its current diastolic configuration (**b**).

The above findings can be best understood mechanistically in relation to the underlying mitral valve fibre architecture. As in all heart valve tissues, MV leaflet directional extensibility is directly determined by the underlying network of collagen fibres^40,41^. The collagen fibres are crimped when unloaded but rapidly straighten and thus stiffen the tissue in systole (Figs. 9, 10). Therefore, the cessation of further changes in total MV leaflet tissue extensibility after 4 weeks suggests this process cannot be explained by tissue remodelling events. As stated above, this hypothesis is supported by a recent study in which the biaxial mechanical response of explanted ovine MV leaflet tissues were examined pre-MI and at 8 weeks post-MI^22^. Taken as a whole, these results are consistent with the observation that while the MV experiences *plastic* deformations in IMR, these are not accompanied by changes in the instrinsic mechanical properties of the underlying collagen fibre network (up to 8 weeks post-MI). Regardless of the exact time course of these processes, our results clearly indicate that the post-MI MV leaflet tissues are no longer normal, which in turn may be another source of repair failures.

From a broader perspective, the results of the present study have several important clinical implications with respect to the treatment of IMR. While IMR is not inherently a valvular disease, but rather a consequence of maladaptive LV remodelling post-MI, the present study is one of the few to elucidate the large extent to which the MV can adapt, either positively or negatively, to substantial changes in its ventricular and haemodynamic environment. The timeline of MV remodelling we have observed in the case of post-MI adaptation is consistent with a previous investigation of MV remodelling in the non-pathological case of pregnancy^42^, in which only passive MV adaptation was observed in early pregnancy, followed by active remodelling of the tissue’s collagen and elastin networks throughout middle and late pregnancy. Together, these studies indicate that MV adaptations to alterations in loading do not occur at the same rate—nor via the same mechanisms—over time, highlighting the complexity of the MV as a biologically responsive tissue. To further elucidate the MV remodelling process, we are currently performing detailed in vitro studies to examine how collagen fibre architecture, tissue composition, and biosynthetic activity in the MV change following MI, which will allow us to draw connections between tissue-level deformations and cellular behaviour in vivo^15^.

The modelling and analysis pipeline we have utilised is not specific to post-MI MV assessment and can be applied to the evaluation of repair-induced MV remodelling as well. The long-term adaptive effects from annuloplasty, which alters MV geometry globally, as well as recently developed leaflet clipping (e.g. MitraClip)^43^ and chord-based (e.g. Neochord)^44^ repair devices, which induce more local stress concentrations, remain largely unknown. The design and refinement of such devices, as well as their proper selection for treating a heterogeneous patient population, would thus greatly benefit from a deeper understanding of the MV remodelling process. Future extensions of this work may also jointly account for the synergy between the MV and LV, since they more precisely behave as a single unit. Insights from the present study can be exploited to improve outcomes of post-MI therapies that primarily target the LV wall affected by MI (e.g. hydrogel injections), for example^45,46^. Moreover, our results hint at the possibility of exploiting patient-specific post-MI MV remodelling assessments to steer not just the design of MV repair techniques themselves, but also the timing of repair surgery. We speculate, for example, that IMR treatment plans accounting for both the current functional state of the MV—which is far from “normal” (i.e. pre-MI)—as well as a rational, evidence-based prediction of how MV remodelling will progress until and after surgery would yield lower rates of IMR recurrence following repair.

While comprehensive, the scope of the present study was limited in several respects. First, our computational method uses image-derived diastolic and systolic geometries of the leaflets only and not the MVCT (which are mostly invisible in rt-3DE). Thus, our ability to predict the post-MI state using only the pre-MI geometry, or similarly to predict the post-repair state using only the pre-repair geometry, is limited. In our ongoing work, however, we are combining this leaflet-based approach with a novel methodology for creating functionally equivalent models of the MVCT, which will enable such predictions given only knowledge of the requisite boundary conditions^33,47^. We note too that the rt-3DE images were collected only over an 8-week period post-MI. Even though our results suggest that at least some of the changes experienced by the MV have already stabilised by 4 weeks, longer term follow-ups will be required to confirm whether the trends observed over this time remain steady, accelerate, or even reverse as a consequence of complex remodelling mechanisms. More comprehensive studies are also necessary to examine the effects of different MI locations as well as repair surgery on the geometry, deformation, and remodelling of regurgitant MVs on a patient-specific basis. Furthermore, the effects of spatial heterogeneity in the MV leaflet tissue’s microstructure, including fibre orientation and alignment as well as constituent volume fractions in different regions of the leaflet, must also be examined more closely, since these aspects of the leaflet’s biomechanics may themselves change post-MI^40,41,48–52^.

An additional consideration is the potential confounding effects of age-related growth on the results. While there was no control group explicitly used, we felt it was unnecessary as the current animal model is very well established for the studies of left ventricular infarction and ischemic mitral valve regurgitation, and has been used extensively by our group for almost three decades. Moreover, as stated in the "Methods" section we used adult Dorset sheep following these well established procedures. In addition, results of ovine MV annular dimensions reported in that were euthanized at the age range that spanned the present study clearly demonstrated no change in annular dimensions with age (private communication). The results of the present study thus accurately represent the dimensional changes in post-MI MV.

The results of our present study shed significant light on the post-MI remodelling behaviour of the MV. These findings are highly relevant to the design of MV repair devices and the optimisation of surgical strategies, as they directly elucidate the the pre-surgical state of the MV. While current repair endeavours largely seek to return the MV to its pre-MI state, our results suggest that this approach may not be favourable, or even possible, especially given the substantial changes in stretch exhibited by the valve. Instead, an effort to place the MV in an alternative state, as close as possible to its homeostatic state, may lead to decreased maladaptive long-term remodelling and thus superior repair outcomes. In our ongoing work, we are building upon the present study to investigate how fibre architecture, tissue composition, and biosynthetic activity in the MV change following MI, which will allow us to draw connections between tissue-level deformations and cellular behaviour in vivo^15,47^. We note too that the present study also has implications for minimally invasive MV repair technologies, such as clips, which induce focal stress concentrations. Such focal effects may lead to long-term remodelling that may limit the durability of such devices, so that patient-specific methodologies may be required to determine optimal design and usage.

## Methods

### Surgical protocol

All animal protocols used in this study were approved by the University of Pennsylvania’s Institutional Animal Care and Use Committee and complied with the National Institute of Health’s guidelines for the care and use of laboratory animals (NIH Publication 85–23, revised 1996). The University of Pennsylvania, with the Supervision of the School of Veterinary Medicine, maintains a full-service vivarium facility operated by the University Laboratory Animal Research (ULAR) organisation. The vivarium is directly adjacent to the laboratory’s operating room suite.

A posterior MI was induced in eight non-diseased adult Dorset sheep, following established procedures that have been previously described in detail^53–55^. Briefly, subjects were anaesthetised with sodium thiopental (10–15 mg/kg intravenously), intubated, and ventilated with isoflurane (1.5–2%) and oxygen. Surface electrocardiogram, arterial blood pressure, and other vital signs were continuously monitored throughout the procedure^55^. Under sterile conditions, subjects underwent a thoracotomy to allow ligation of the second and third obtuse marginal branches of the circumflex coronary artery. Permanent occlusion of these arteries reliably results in a transmural posterior MI that involves approximately 20% of the LV mass, includes the entire posterior PM, and causes a gradual onset of severe IMR within 8 weeks^53,55^. After haemodynamic and electrophysiological stabilisation, the incisions were closed and subjects were allowed to recover for subsequent follow-up imaging.

### Data collection and geometric modelling

**Figure 11:**
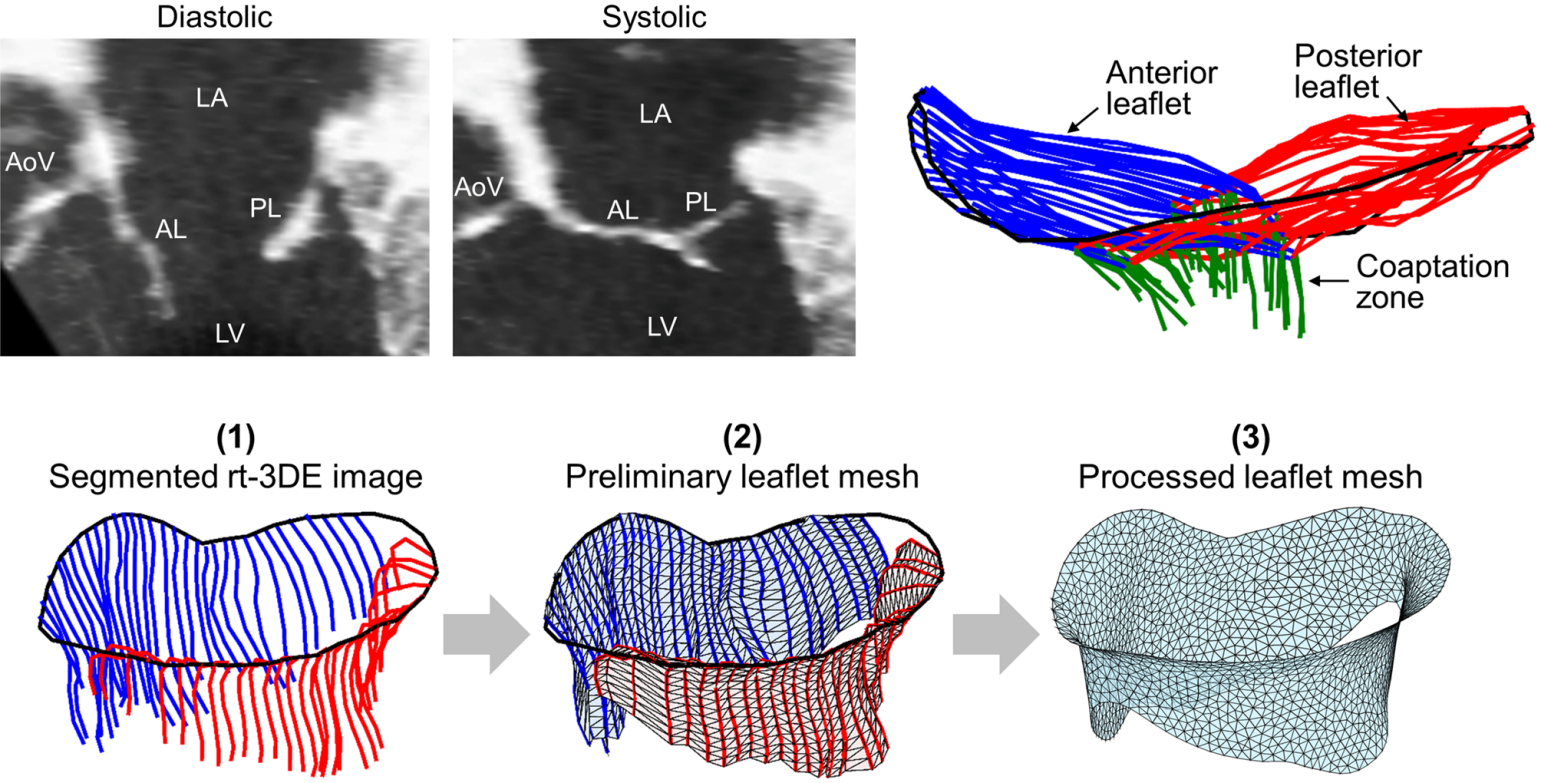
Pipeline for MV geometric modelling, starting from noninvasive rt-3DE images of the valve. Cross sections of each image are first interactively traced, and then the segmented geometry is converted to a subject-specific triangulated mesh suitable for FE simulations (adapted from Rego et al.^20^). AL, anterior leaflet; PL, posterior leaflet; LA, left atrium; LV, left ventricle; AoV, aortic valve.

Following established protocols^56,57^, electrocardiographically gated full-volume images were acquired over four consecutive cardiac cycles, with an imaging depth of 12–16 cm. From each subject’s data series, representative rt-3DE images of the MV in the end-diastolic and end-systolic states were selected for analysis and exported in Cartesian format with an approximate isotropic resolution of 0.6–0.8 mm. Each specimen was imaged pre-MI, immediately post-MI ($t = 0$ weeks), 4 weeks post-MI ($t = 4$ weeks), and 8 weeks post-MI ($t = 8$ week). Next, from each rt-3DE image, the plane of the MV orifice was rotated into a short-axis view, and the geometric centre of the orifice was translated to the intersection of two long-axis planes corresponding to the intercommissural and septolateral axes of the MV orifice (Fig. 11, top). A rotational template consisting of 18 long-axis cross-sectional planes separated by 10$^\circ$ increments was superimposed on the rt-3DE image. Two annular points intersecting each of the rotational planes were then identified by means of orthogonal visualisation of each plane and marked interactively. The anterior and posterior leaflets were then traced separately in parallel long-axis cross sections, 1 mm apart and sufficient to span the entire MV from commissure to commissure. For systolic images, the coaptation zone between the two leaflets was also independently labelled in each parallel cross section (Fig. 11, top).

Starting from the segmented pre-MI end-diastolic image of each MV, we constructed subject-specific meshed geometries of the entire leaflet surface, including both the annular and leaflet free-edge boundaries. First, the interactively traced cross sections of the leaflet medial surface were all parameterised using shape-preserving piecewise cubic interpolating polynomial curves^58^, and re-discretised with an equal number of spline segments of uniform arc length^59^. It is straightforward from this representation to obtain separate preliminary meshes of the anterior and posterior leaflets, using a 2D Delaunay triangulation scheme (Fig. 11, bottom). To join the two leaflets in the commissural regions, circumferential cross sections of the entire leaflet surface were generated using a cubic spline interpolation of corresponding parametric (i.e. relative arc length) locations on each trace curve, with periodic tangent and curvature boundary conditions used to preserve surface smoothness. This procedure yielded a fully parameterised spline representation of the leaflet surface. We then discretised the surface uniformly using a Poisson-disk sampling method^60^, tuning the sampling density to arrive at a 1-mm spatial resolution^32^. Lastly, we reconstructed the surface from the new sample points using a ball-pivoting algorithm^61^, and smoothed the resulting mesh using Taubin’s method^62,63^. The end product of this processing step was a complete subject-specific leaflet geometry for each specimen (Fig. 11, bottom). Circumferential and radial material directions were then defined as in Rego et al.^20^

### Image-based stretch estimation

**Figure 12:**
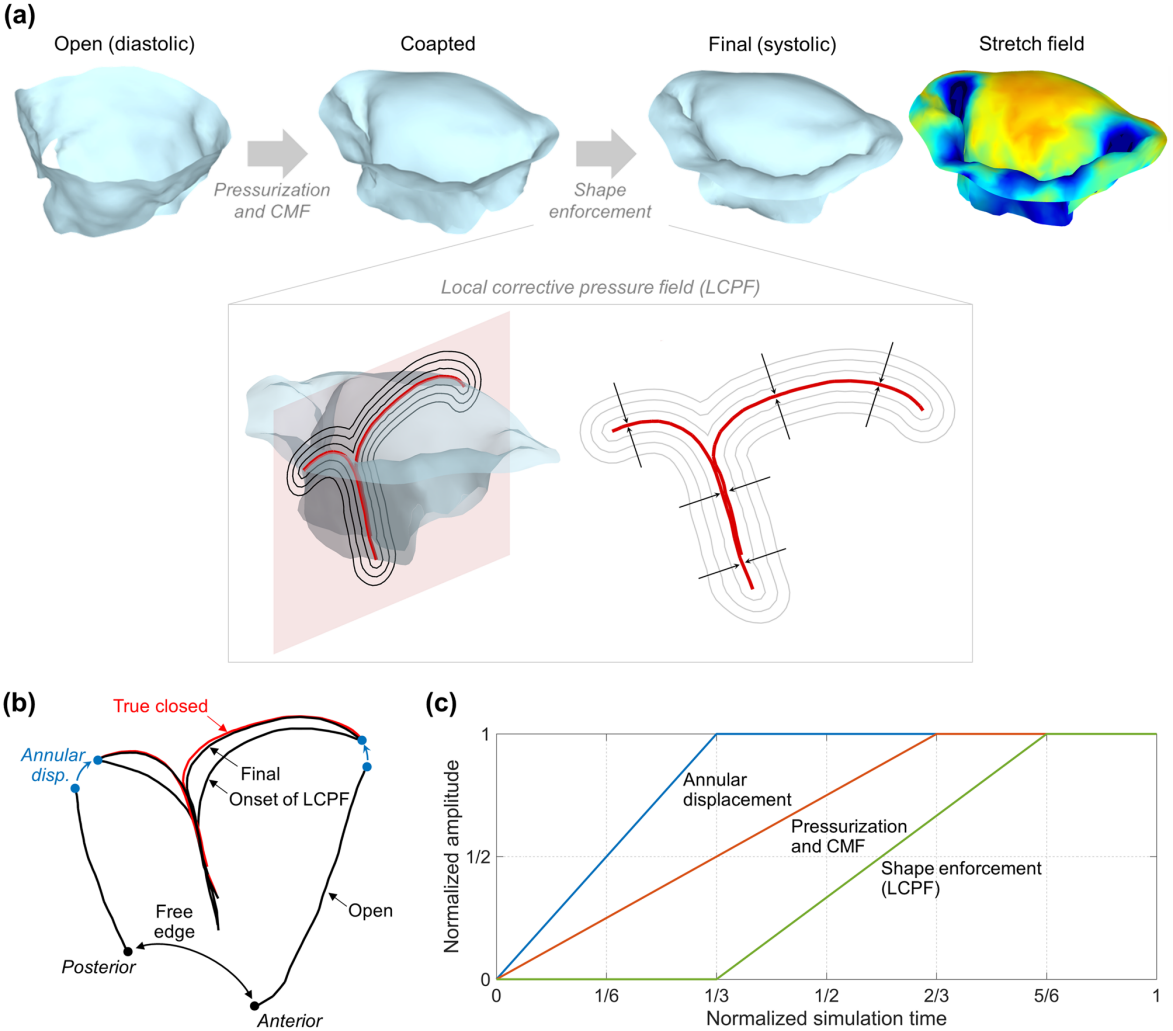
(**a**) Snapshots of a simulated MV throughout a FE closure simulation, showing initial coaptation by pressurisation followed by shape enforcement using the LCPF, resulting in a locally computed stretch field. The LCPF causes the MV mesh to be pushed locally and orthogonally toward the imaged final configuration, thus enforcing the overall shape without biasing local stretch estimates (see Rego et al.^20^ for details). (**b**) Snapshots of a cross section of the simulated mesh, shown for the same time points as in (**a**); a cross section of the true closed shape (red) is shown for comparison with the final configuration. (**c**) Coordination of the boundary conditions and loads that are applied during the FE simulation to ensure proper closure and shape enforcement (adapted from Rego et al.^20^).

To determine systolic deformation fields across the entire MV leaflet surface noninvasively, we utilised a previously validated image-based stretch estimation method developed by our group^20^, which yields local stretch information directly from clinical-quality in vivo images (Fig. 12). Essential features of our approach are that it does not require knowledge of the MV chordal structure nor exact mechanical properties, which cannot be extracted from rt-3DE. Moreover, our method does not rely on physical markers to extract surface deformations; we thus did not require any material point correspondence between open-state and closed-state images when estimating systolic stretches. Instead, we exploited the fact that the gross subject-specific closed-state geometry of the leaflets can be precisely acquired from systolic scans, and developed the following method to enforce this closed shape during a finite element (FE) simulation of MV closure: While pressurising the open-state valve mesh, we used a downward chord-mimicking force (CMF) to prevent prolapse and penalised any mismatch between the simulated and true (i.e. imaged) closed shapes of the leaflets using a local corrective pressure field (LCPF), which was at any instance and location linearly proportional to the shortest distance between the FE mesh and the true MV medial surface (Fig. 12). In this way, the LCPF locally “pushes” regions of the MV so that the imaged closed-state geometry was matched.

To estimate stretches between two subject-specific diastolic scans (from different time points), the same stretch estimation method was applied, only without atrioventricular pressurisation. To allow for both biomechanical and clinical interpretations, all systolic stretch fields were then expressed with respect to both pre-MI and current diastolic states.

### Post-processing and analysis

**Figure 13:**
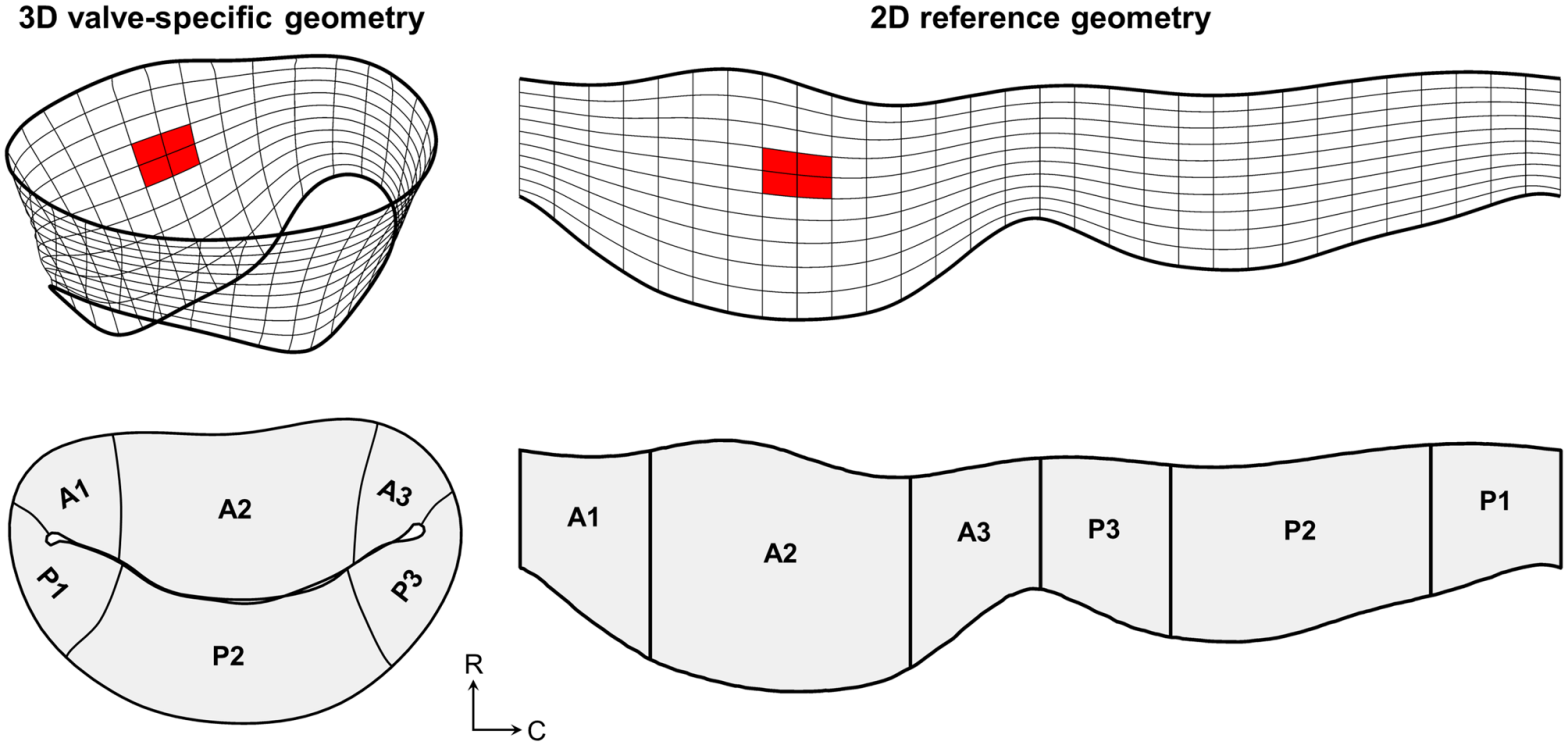
A representative MV, parameterised using a conformal map to allow for precise inter-subject registration of local leaflet attributes (e.g. directional stretches) as well as systematic definition of Carpentier leaflet segments. The parameterised 3D surface can be naturally unfolded onto a 2D domain for visualisation of attributes in otherwise obscured regions of the leaflet (see Supplementary Information for stretch field results in 2D).

To detect statistically significant changes in regional stretches, it was necessary to express the computed stretch fields in a way that allowed for direct registration of local stretch values between different MV specimens. To accomplish this, we parameterised each subject’s MV leaflet geometry using a conformal (i.e. angle-preserving) map, which was obtained by solving the Laplace equation on each triangulated mesh with Dirichlet boundary conditions on the annulus and free edge^64^. The use of a conformal map was crucial to our approach, as it preserved orthogonality between the circumferential and radial directions. The stretch fields of each subject were then defined in the parametric space, which registered the stretch values from each original 3D valve geometry to a 2D domain with orthogonal coordinates that were correspondent across all specimens (Fig. 13). The ability to compare locally correspondent stretch values between subjects and time points enabled the averaging and statistical analysis of the obtained stretch results, for which we used paired $t$-tests due to the longitudinal subject-specific data acquired. Additionally, this parameterisation technique allowed for the systematic definition of leaflet segments based on parametric locations for each MV geometry, according to the conventional Carpentier nomenclature (A1–A3 for anterior leaflet, P1–P3 for posterior leaflet)^65^.

## Supplementary Information

Supplementary Information.

## Data Availability

The data sets generated during and/or analysed during the present study are available from the corresponding author on reasonable request.
