# Supplementary material for: In vivo assessment of mitral valve leaflet remodelling following myocardial infarction
Source: Sci Rep. 2022 Oct 26;12:18012. doi: 10.1038/s41598-022-22790-0 (PMC9606267; doi:10.1038/s41598-022-22790-0)
Supplement: Supplementary file 1 — Supplementary Information. [file 41598_2022_22790_MOESM1_ESM.pdf]

SUPPLEMENTARY INFORMATION

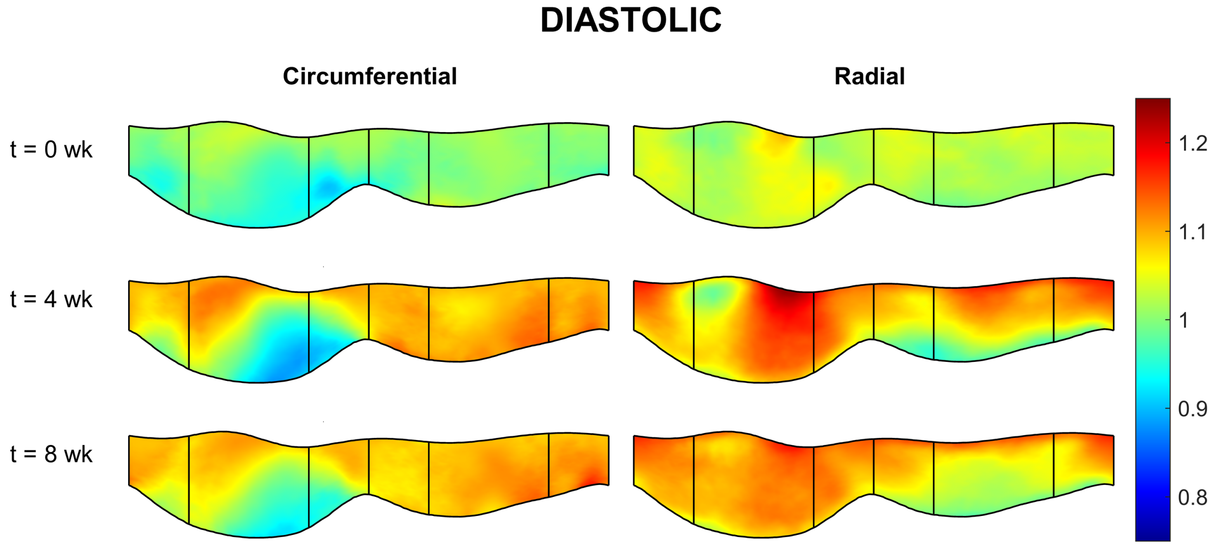

Figure S1. Diastolic directional stretches, referenced to the pre-MI diastolic configuration, shown on a 2D reference geometry with delineated leaflet segments. Definition of the associated leaflet segments indicated are shown in **Figure 13**.

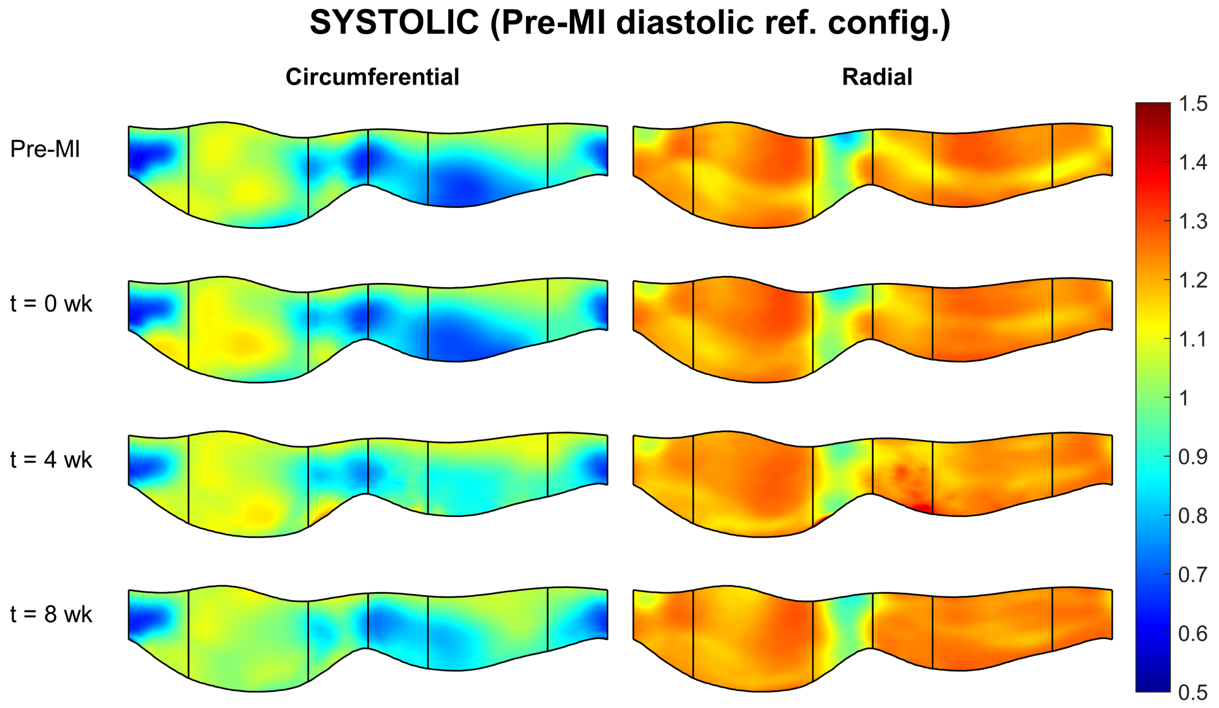

Figure S2. Systolic directional stretches, referenced to the pre-MI diastolic configuration, shown on a 2D reference geometry with delineated leaflet segments. Definition of the associated leaflet segments indicated are shown in **Figure 13**.

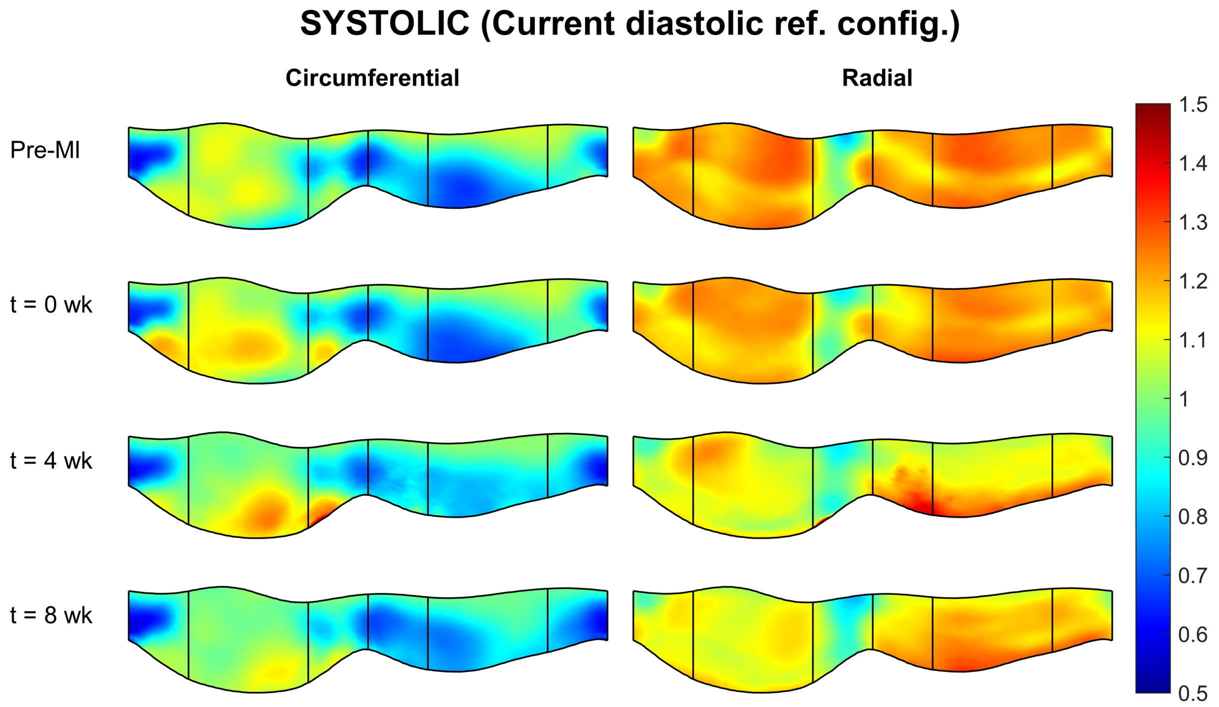

Figure S3. Systolic directional stretches, referenced to the current time point's diastolic configuration, shown on a 2D reference geometry with delineated leaflet segments. Definition of the associated leaflet segments indicated are shown in **Figure 13**.

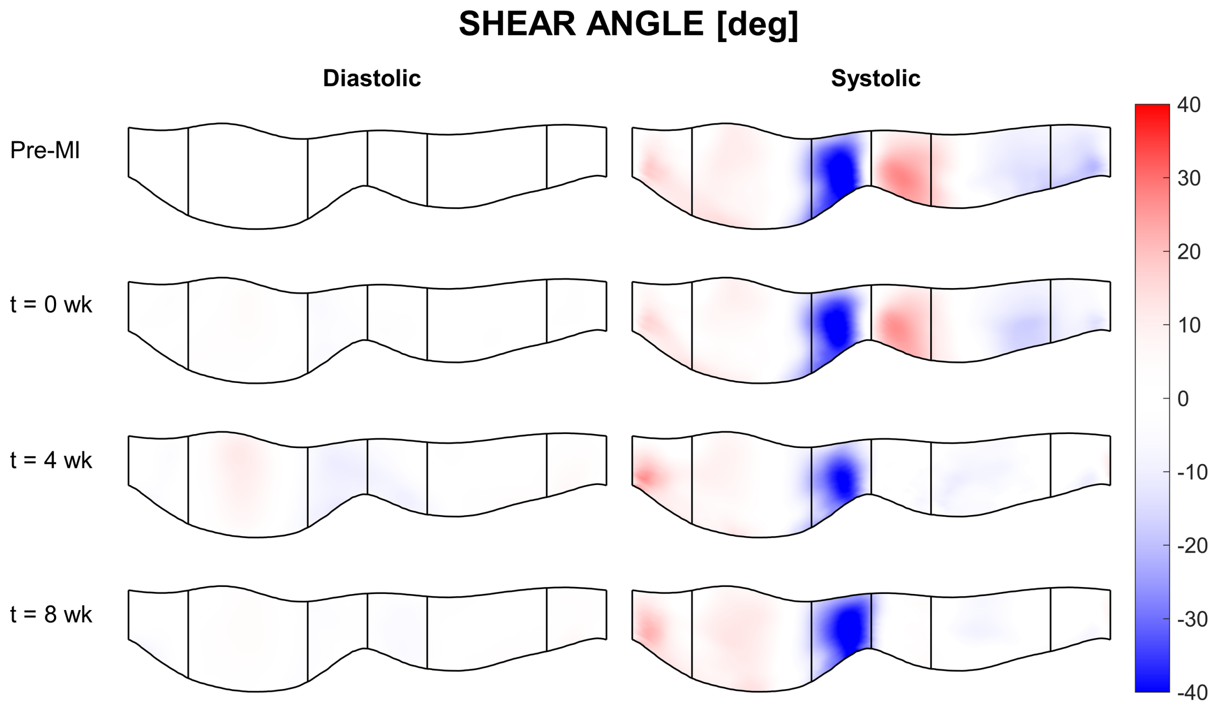

Figure S4. Diastolic and systolic shear angle, shown on a 2D reference geometry with delineated leaflet segments.
